# The Reciprocal Relationship between Suicidality and Stigma

**DOI:** 10.3389/fpsyt.2017.00035

**Published:** 2017-03-08

**Authors:** Bernardo Carpiniello, Federica Pinna

**Affiliations:** ^1^Department of Medical Sciences and Public Health, University of Cagliari and Psychiatric Clinic, University Hospital, Cagliari, Italy

**Keywords:** stigma, internalized stigma, suicide, mental illness, mental disorders, risk factors, media

## Abstract

**Introduction:**

Although suicidality is frequently the cause of stigma, it is conversely true that stigma may be the cause of suicidality. The present paper focuses on the complex relationships that exist between suicidal behavior and stigmatizing attitudes.

**Methods:**

A narrative review of the topic will be presented on the basis of the relevant literature collected from an electronic search of PubMed, ISI Web of Knowledge, and Scopus databases, using stigma, public stigma, structural stigma, perceived stigma, self-stigma, suicide, attempted suicide, and suicidality as key words.

**Results:**

A negative perception is frequently held of suicidal people, labeling them as weak and unable to cope with their problems, or selfish. Individuals who have attempted suicide are subject to similar processes of stigmatization and “social distancing”; insurance policies include an exclusion clause against death by suicide. Subjects with a direct personal experience of depression or suicide strongly endorse a feeling of self-stigma; those who have attempted suicide are often ashamed and embarrassed by their behavior and tend to hide the occurrence as much as possible. Similar processes are observed among family members of subjects who have committed suicide or made a suicide attempt, with a higher perceived stigma present in those bereaved by suicide. Perceived or internalized stigma produced by mental or physical disorders, or through belonging to a minority group, may represent a significant risk factor for suicide, being severely distressing, reducing self-esteem and acting as a barrier in help-seeking behaviors.

**Conclusion:**

With the aim of preventing suicide, greater efforts should be made to combat the persisting stigmatizing attitudes displayed toward mental disorders and suicide itself. Indeed, the role of stigma as a risk factor for suicide should further motivate and spur more concerted efforts to combat public stigma and support those suffering from perceived or internalized stigma. Experts and scientific societies should form an alliance with the media in an effort to promote a marked change in the societal perception of mental health issues and suicide. As stigma may result in severe consequences, specialist care and psychological interventions should be provided to populations submitted to stigma.

## Introduction

Suicide is one of the major public health concerns worldwide, currently listed as the 15th most common cause of death, and accounting for approximately 1.4% of all mortalities; more than 800,000 people die due to suicide, with even higher number of suicide attempts each year (1).

Accordingly, particular consideration should be given to suicide, not only in view of its epidemiological relevance, but particularly as it is one of the human behaviors and conditions at highest risk of stigmatization, on a par with mental disorders, with which suicide is generally associated. Indeed, as reflected in media depictions (2), in the public opinion suicide is largely associated with mental illness; however, average estimates of psychiatric disorders among suicide victims vary from 69.9 to 88.2% in North America and 90.4% in South Asia (3), with a substantial proportion of suicidal cases lacking any association with mental disorders, including subthreshold conditions (4). Although the situation has changed somewhat in recent years, stigmatizing attitudes toward suicide still persist, implying a series of relevant consequences for survivors and their families. Moreover, irrespective of how stigma is determined (suicidality itself and/or mental illness, somatic illness, being part of a minority), it should be viewed as a potent stressor (5), capable of constituting *per se* a risk factor for suicide. Based on these premises, this narrative review aims to focus on the reciprocal relationship between suicidality and stigma and its consequences. After a brief analysis of the historical and religious origins of negative attitudes toward suicide, the review examines the current literature on suicidality as a cause of stigma, both in terms of the nature and extent of stigmatizing attitudes and consequences for suicidal persons and their families. The issue of stigma as a risk factor for suicidality is then considered. Finally, lessons to be drawn from the current literature and problems to be faced are discussed.

## Methods

**Figure 1:**
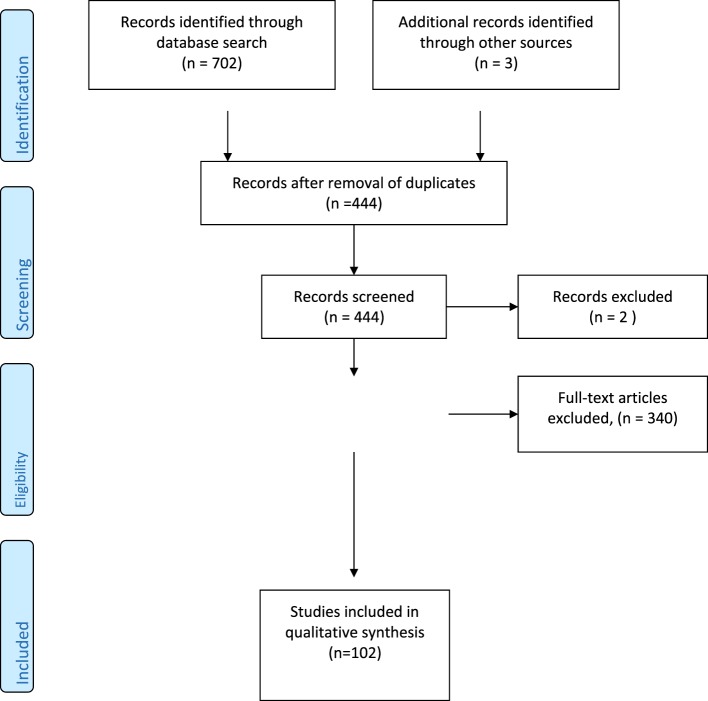
**Literature search flow diagram**.

We performed an electronic search of PubMed, ISI Web of Knowledge, and Scopus databases, without any restriction as regard to time and language, using stigma (i.e., the set of beliefs and attitudes that induce people to refuse, stave off, or fear people perceived as being “different”), religion, public stigma (i.e., the prejudice and discrimination endorsed by the general population), structural stigma (i.e., the set of those practices, regulations or rules, policies of a given social institution in order to restrict the rights and/or opportunities of citizens affected by a mental disorder), perceived stigma (i.e., the discrimination and devaluation by others as perceived by subjects), self-stigma (i.e., the negative public attitudes internalized by people suffering from mental problems), suicide, attempted suicide, and suicidality as key words (Figure 1). Only published full papers were examined, including original researches, reviews, and position papers. References found in selected papers were checked in order to identify other papers that could be considered as potentially relevant. Finally, in the case of research contributions, papers were considered on the basis of pertinence of their results to the topics of this narrative review and of their relevance and/or comprehensiveness in the case of reviews or position papers.

## The Historical and Religious Origins of Negative Attitudes toward Suicide

Suicide was tolerated by the Greeks and Romans, although considered by Aristotle to be a detriment to the economy and a means of irritating the Gods (6). In the Judeo–Christian tradition, there seems to be no trace of negative attitudes toward suicide (7), and the Bible itself contains no trace of condemnation of suicide (8). Since the times of St. Augustine, who viewed suicide as being incompatible with Christian values, the Catholic Church has condemned suicide, ultimately resulting in the excommunication of suicides and their family members, burial in unhallowed ground, and confiscation of property; this attitude has however gradually been attenuated during the twentieth and twenty-first centuries (9). Indeed, the Compendium of Catechism of the Catholic Church stated not only that “Everyone is responsible for his life before God who has given it to him. It is God who remains the sovereign Master of life. We are obliged to accept life gratefully and preserve it for his honor and the salvation of our souls. We are stewards, not owners, of the life God has entrusted to us. It is not ours to dispose of,” but also affirms that “the voluntary cooperation of suicide is contrary to the moral law. Grave psychological disturbances, anguish, or grave fear of trial, suffering, or torture can diminish the responsibility of the one committing suicide. We should not despair of the eternal salvation of persons who have taken their own lives. God, through ways known to Him alone, can provide the opportunity for salutary repentance. The Church prays for persons who have taken their own lives” (10).

Among other Monotheisms, Islam adopts an attitude of condemnation of suicide (7), in the same way as Judaism, which considers the value of human life as supreme (11). Buddhism and Hinduism hold no traditionally negative view of suicide (7), although discordant voices have been raised to this regard (12). Whatever the importance of the different positions held by some religious faiths toward suicidal behavior, they seem to have a certain value in relation to suicide prevention, given that adherence to the norms and moral values dictated by religious beliefs has been associated with a lower risk of suicidality in different clinical conditions (13, 14).

## Stigma toward Suicide

Notwithstanding the apparent slight decrease of stigma toward people affected by mental disorders people over the recent years, no similar reduction of the stigma associated with suicide and suicide-survivorship has been reported (15); limited data however appear to show a trend toward the reduction of suicide-associated stigma, particularly with regard to moral disapproval (16). However, prejudicial attitudes toward those who commit suicide are still quite common, as highlighted in recent studies conducted in a series of very different sociocultural contexts using largely heterogeneous designs and methods. An Australian survey conducted on a sample of 676 subjects showed that between 30 and 40% respondents considered suicide as a punitive, selfish, offensive, or reckless act; a 20–30% proportion of the sample saw suicide as a sign of weakness or a thoughtless, irresponsible, cowardly, senseless, or attention-seeking act (17). Suicidal people are generally labeled in a negative sense, as being weak and unable to cope with their problems, or selfish (18). A recent Canadian survey among an adult population regarding stigmatizing attitudes and beliefs about male depression and suicide show how more than a third of responders agreed that men affected by depression are unpredictable, among those who had no direct experience of depression or suicide; overall, more males expressed stigmatizing opinions about depression respect to females; a greater proportion of female respondents endorsed items indicating that men who commit suicide are disconnected, lost, and lonely (19). A large Australian online survey relating to attitudes toward suicide revealed how a lower degree of exposure to suicide, older age, male gender, less education, and culturally diverse backgrounds was associated with poorer knowledge; conversely, stigmatizing attitudes were associated with male gender, younger age, and culturally diverse backgrounds (20). A Korean nationwide community study of factors related to social stigma of people with a history of suicide, suicide attempts, or depression revealed that older men with lower education and no history of previous suicide attempts predicted the degree of stigmatizing attitudes toward people attempting suicide (21). A recent Chinese paper investigating users’ attitudes toward suicide attempts broadcasted on social media (Weibo) showed how more than 33% of posts could be considered as “stigmatizing”; among these, post codes as “deceitful,” “pathetic,” and “stupid” were, respectively, 22, 16, and 15% (22). Participants randomly assigned to read one of the three fictional obituaries, identical except for the reported cause of death (suicide, cancer, or drug overdose), viewed people listed as succumbing to death by suicide more negatively than those who had died from cancer (23).

Public stigma toward suicide moves parallel to the problem of perceived stigma for both those who have attempted suicide and by the family members of people who have committed or attempted suicide. Data from focus groups comprising a series of diverse stakeholders (suicide attempt survivors, family members of people who have died by suicide and suicide loss therapists) set up to qualitatively investigate suicide stigma revealed an array of stereotypes, prejudices, and discrimination, in particular the fact that those who attempted suicide are predominantly viewed as attention-seeking, selfish, incompetent, emotionally weak, and immoral (24). In a recent study of individuals with a lifetime history of suicidal behavior recruited through the American Association of Suicidology, respondents reported the highest rates of perceived stigma from a close family member (57.1%) and emergency department personnel (56.6%); the results of the study revealed how subjects who had displayed previous suicidal behavior were more likely to experience stigma, particularly from non-mental health providers and members of their social network (25). Suicide bereavement is perceived differently from natural loss, at times producing a deeply profound effect on the family, friends, and associates of the victim, which goes beyond the suffering inflicted by the immediate loss; indeed, one of the discriminating elements observed in suicide bereavement is the stigma experienced by survivors (26). A cross-sectional study of about 3,400 respondents who had suffered a sudden bereavement (i.e., the death of someone close from the age of 10 years) demonstrated how people bereaved by suicide displayed higher perceived stigma than those bereaved by sudden natural death and people bereaved by other sudden unnatural deaths (27).

## Consequences of Stigma toward Suicide

Discrimination is a behavioral response to prejudice and is essentially dependant on the type of emotional reaction associated with the latter. Discrimination may be direct, as shown by studies that demonstrate how people who have attempted suicide are subject to similar processes of “social distancing” as those directed at ethnic or religious minorities (28, 29). Suicides typically leave a total of six or more survivors, with a consequent long-lasting emotional turmoil, which may in some cases end with the survivors’ own suicide (30). Family members of individuals who have committed suicide are often judged and blamed for their relatives death (31), with post suicidal bereavement being burdened by the complex psychological impact of the suicide on those close to the victim, fostered by a societal perception that self-given death is considered a failure for those who die by suicide and the family, and by the society blaming the survivors for their losses (26). Indeed, significantly higher feelings of shame, and an increased sense of responsibility and guilt are commonly found in those bereaved by suicide, both in comparison with bereavement by a sudden natural and unnatural death (27).

Structural discrimination comprises the negative consequences resulting from imbalances and injustices experienced in terms of use of facilities and social services, political decisions, and legislation. With specific regard to suicide, probably the most impressive form of discrimination is represented by the fact that throughout the majority of industrialized countries, insurance policies include an exclusion clause against death by suicide and those deemed to be at risk of suicide will not be able to obtain life insurance (32). People with direct personal experience of depression or suicide, both male and female, strongly endorse stigmatizing attitudes toward themselves (18). Self-stigma (or internalized stigma), a self-discrimination process that suicidal individuals (and/or their families) put into effect, is of paramount importance. Subjects with a direct personal experience of depression or suicide, both male and female, strongly endorse a feeling of self-stigma, while those who have attempted suicide are often ashamed and embarrassed by their behavior and tend to conceal the occurrence as much as possible (33–35). Similar processes are observed among family members of individuals who have attempted suicide (26, 31).

## Stigma as a Risk Factor for Suicide

Psychological distress due to stigmatizing attitudes may be an exceedingly severe burden and at times result in extreme consequences. Exposure to suicide in someone close has been found to be associated with a series of negative health and social outcomes, including an increased rate of suicide among partners and mothers of people who died by suicide, a more pronounced recourse to psychiatric care by parents bereaved by the self-given death of an offspring, and a higher risk of depression in offspring of parents who had committed suicide (36). However, although adults bereaved by suicide had a higher probability of attempting suicide than those bereaved by sudden natural causes, the significant association between bereavement by suicide and suicide attempt became non-significant when adding perceived stigma, a finding which could be interpreted as indicating stigma as a marker for motivational moderators of suicidality after a negative life event, such as reluctance to seek help, thwarted belongingness or perceived burdensomeness (37). In addition to those specifically relating to suicidality, other forms of stigma should be taken into account due to the severe psychological distress they may cause. Indeed, mental disorders are likely the public health issue featuring the strongest link between stigma and suicide. Indeed, perceived stigma is one of the main factors associated with the risk of suicide among the mentally ill, with suicide at times being seen as a means of escaping from the stigma itself (38, 39). Furthermore, stigma is a highly relevant factor related to suicide among people affected by schizophrenia and other mental disorders (38, 40–46), including people considered at risk of psychosis (47). Confirmation of the link between stigma toward the mentally ill and suicide has been provided by a recent European study (48) showing that age-standardized suicide rates were negatively correlated (beta = −0.46, *p* = 0.014) with levels of social acceptance; these findings were derived by crossing data for the indicators of social acceptance of people with mental health problems (Eurobarometer data) and data on suicide rates and socioeconomic indicators (Eurostat) obtained from 25 EU countries for the year 2010. Evidence from this study has been interpreted as implying that stigma contributes toward explaining the risk of suicide and differences in suicide rates detected throughout the different nations; according to the authors, the link between stigma and suicide rates could be explained by a series of assumptions, including the role of stigma as a stressor and cause of social isolation (48). Together with other consequences of stigma such as unemployment, and subjective experiences such as hopelessness, the latter constitutes an important risk factor for suicide among the mentally ill (49). Discrimination is seemingly experienced as a stressor that exceeds coping resources of those who are stigmatized; moreover, this leads to develop a negative self-image, with the perception of a lack of support by their own social networks; as a consequence, the increased anticipation of future negative events together with the perceived absence of social support may lead the mentally ill into hopelessness and suicidality (43). However, research findings have demonstrated how, in addition to discrimination and perceived stigma, other factors may also contribute toward explaining the rather intricate link between stigma and suicide among people suffering from mental disorders. Indeed, a longitudinal study has highlighted how self-stigma (or internalized stigma) is strongly related to suicidality (50). Levels of insight, depression, and internalized stigma may be associated, at least in schizophrenia, with a higher suicide risk (51). Suicidality related to self-perceived and internalized stigma may be explained by the mediating role of low self-esteem (52, 53). Increased self-labeling as “mentally ill” has been found to be associated with suicidality, being directly and indirectly mediated by social isolation, which in turn is associated with low self-estem (54). Additional information relating to the intriguing link between stigma and suicidality has been gathered from a large community sample examined by means of interviews and self-reports, with the aim of collecting information on perceived stigma, secrecy, hopelessness, and suicidal ideation. Participants who had previously been referred to mental health services were labeled as “mentally ill,” with the stigma attached to mental illness contributing toward suicidal ideation in these people; one possible explanation for this association is the relation between perceived stigma and secrecy, which seems to introduce particular negative emotional consequences (55).

Some authors (45) have also hypothesized a “direct” mechanism whereby perceived stigma acts as a “barrier” to accessing mental health services, which should be considered together with the “indirect” mechanism relating to self-stigma. Indeed, one of the most widely accredited explanations of increased suicidality due to stigma is its influence on help-seeking behavior. In a national survey conducted on a sample of the Australian population who were assessed by means of case vignettes depicting depression with or without suicidal intent, the presence of high levels of personal stigma among respondents was a strong, independent predictor of the opinion that depression, both associated and not associated with suicidal thoughts, should best be coped with alone (56). In a web survey of a large sample of medical students from a US university, approximately 14% were found to be affected by depressive disorders ranging from moderate to severe. In particular, throughout the last 2 years of their studies, students reported suicidal thoughts (7.9%); these students indicated statistically more frequent fears of stigmatization (57).

An extensive survey of medical students in the USA revealed that a third of respondents had sought help for a mental health problem over the previous year; respondents with high levels of distress were found to be more likely to agree or strongly agree with 8 of 10 perceived stigma items (58) compared to students who were not distressed. Forty-four percent of college students with a lifetime history of suicidal ideation failed to seek treatment during young adulthood; ambivalence about treatment need or effectiveness, fear of stigma, and financial concerns were found to be the most relevant barriers to treatment (59). Finally, a large survey of undergraduate and graduate students from 15 US Universities demonstrated that correlates of help-seeking and treatment use among individuals referring serious suicidal thoughts over previous years were perceived need, beliefs about treatment effectiveness, contacts with service users, personal stigma and perceived stigma, level of social support, belonging to minorities and ethnicity (60). Although the majority of studies investigating young people highlight the relevance of stigma as a barrier to help seeking, some studies debunk this role. Indeed, a survey conducted among college students who did not seek treatment and deemed to be at high risk for suicide, the most commonly reported barriers included perception that treatment was not needed (68%) lack of time (26.8%) and preference for self-management (18%), while stigma was mentioned by only 12% of students (61); attempting to explain the reasons of these somewhat surprising results, the authors hypothesize that it was possible that students were concerned less about stigma than expected because of ongoing efforts aimed at reducing mental health stigma on college campuses, or that other reasons were simply more salient for these students’ help-seeking decisions, particularly when asked to self generate reasons for not seeking help, rather than selecting from an available list of reasons. Another possibility taken into account was that stigma, although not mentioned explicitly, underlies some of the other barriers noted by students, such as not considering problems as warranting professional treatment or preference for self-management, which might reflect an underlying concern about stigma.

Anyway, the role of a reduced help-seeking behavior and fear of stigma among suicidal people, particularly those affected by a mental disorder, has been confirmed by several other studies. Fifty-five percent of people who commit suicide had had no contact with their GP over the previous month, and 68% of suicidal people had not had any contact with mental health services in the last year (62). More than 70% of people with mental disorders fail to seek help or do so very late due largely to non-recognition of having a mental disorder, poor access to care, fear of prejudice, and expectation of being discriminated as people with mental disorders (63). The importance of stigma in reducing help-seeking behavior is particularly consistent in those at higher risk of suicide: only 39% of people at risk had sought help of any kind in the preceding year with the main reasons for not requiring help being, in order: failure to recognize the need for help, the belief that any intervention would be ineffective, and fear of being stigmatized (64). Social, economical, and cultural factors may impinge on help-seeking behavior and subsequently affect suicide rates: 56% of people at risk in high-income countries and only 17% in low-income countries had sought help in the year preceding suicide (63); one study conducted in the Netherlands (65), where suicide rates are quite low, demonstrated an increased openness to calls for help in the presence of psychological problems, a reduced sense of shame and lesser fear of stigma compared to the Flemish population, characterized by higher rates of suicide. Moreover, the levels of perceived stigma were found to be negatively correlated with the propensity to seek informal help in both countries, and with the propensity to seek professional help in Flanders (65). A survey of Asian-American women with a history of depression and suicidality highlighted that the underutilization of mental health services was clearly correlated to cultural factors such as Asian family stigmatizing attitudes and Asian community contribution to mental health stigma (66). Although the data present in literature support the presence of a credible link between stigma of mental illness and suicide, the limitations of some current studies should be taken into account. These limitations include the cross-sectional nature of the large majority of studies, thus not allowing any firm conclusions to be reached as to the direction of causality between stigma, symptoms, and suicidality, in addition to the fact that as suicide is a relatively rare event, many studies have merely regarded suicidal ideation or suicidal attempts as a proxy (49).

It would be a mistake to confine the role of stigma as a risk factor for suicide to individuals with mental disorders, as the fear of stigma extends to people suffering from somatic disorders or who belong to a minority group. These groups are often burdened by harsh labeling attitudes and discrimination, resulting in an increased risk of suicidality. Indeed, lifetime risk of suicidal ideation and attempts is strongly correlated to perceived discrimination among immigrants, as demonstrated in Hispanic people in the USA (67). Moreover, stigma is one of the major risk factors for depression and suicidality among sexual minorities (68–87) and in patients affected by AIDS (88–91) and obesity (92–94).

## Conclusion

The main findings emerging from our paper should be read in light of the well-known limitations of narrative reviews, which are mainly due to certain subjectivity respect to other forms of review as regard to the selection of studies (selection bias due to subjective weighing of studies included in the review and to the lack of specificity in inclusion criteria), the method of study analysis chosen and the possibility of misleading conclusions drawn from the studies considered due to a scarce consideration of relationships between the study characteristics and the results, the difficulties in integrating data derived from large set of studies taken into account (95). Even considering these intrinsic limits, some relevant aspects emerge from our review. First of all, a reciprocal relationship exists between stigma and suicide: suicide may cause stigmatizing attitudes, but stigma toward mental disorders may be a risk factor for suicidality. Both suicide and mental disorders are still today burdened by relevant negative attitudes that can only be tackled by a marked change in societal perception of these issues. Although modest, there is evidence for the effectiveness of antistigma interventions in terms of increasing knowledge and reducing stigmatizing attitudes, at least in the short term (96, 97). A better suicide literacy and low stigmatizing attitudes toward suicide were found to be associated with more pronounced help-seeking attitudes (98), while suicide literacy and stigma reduction programs would benefit community members (99). Mass media interventions may reduce prejudice toward mental disorders, although there is insufficient evidence to determine their effects on discrimination (100); moreover, media may play a significant role in suicide prevention (101). Thus, structured and permanent forms of partnership should be set up between experts, scientific societies, and the media to promote extensive educational efforts aimed at providing deeper insight into mental health issues and thus helping to reduce stigma. The stigma displayed toward suicide may result in severe consequences for people who have attempted suicide or who have been bereaved by suicide; specialist care and specific psychological interventions should be provided to these populations. The role of stigma as a risk factor for suicide should further motivate and spur more concerted efforts aimed at combating public stigma, sustaining self-esteem, reducing isolation and empowering those suffering from perceived or internalized stigma.

## Author Contributions

BC and FP contributed equally to the search for relevant literature and to writing the paper.

## Conflict of Interest Statement

The authors declare that the research was conducted in the absence of any commercial or financial relationships that could be construed as a potential conflict of interest.

## References

[1] WHO. Preventing Suicide – A Global Imperative. (2014). Available from: http://apps.who.int/iris/bitstream/10665/131056/1/9789241564779_eng.pdf?ua=

[2] Carpiniello B Girau R Orrù MG Mass-media, violence and mental illness. Evidence from some Italian newspaper. Epidemiol Psichiatr Soc (2007) 16:251–5. 10.1017/S1121189X00002359 18020199

[3] Cho SE Na SK Cho SJ Im JS Kang SG. Geographical and temporal variations in the prevalence of mental disorders in suicide: systematic review and meta-analysis. J Affect Disord (2016) 190:704–13. 10.1016/j.jad.2015.11.008 26600412

[4] Milner A Sveticic J De Leo D. Suicide in the absence of mental disorder? A review of psychological autopsy studies across countries. Int J Soc Psychiatry (2013) 59:545.54. 10.1177/0020764012444259 22582346

[5] Link BG Phelan JC Stigma and its public health implications. Lancet (2006) 367:528–9. 10.1016/S0140-6736(06)68184-1 16473129

[6] Alvarez A The Savage of God: A Study of Suicide. New York: WW Norton (1990).

[7] Tadros G Hotoff M The stigma of suicide. Br J Psychiatry (2001) 179:178 10.1192/bjp.179.2.178 11483487

[8] Barraclough GM The bible suicides. Acta Psychiatr Scand (1992) 86:64–9. 10.1111/j.1600-0447.1992.tb03228.x 1414404

[9] Pritchard C Suicide. The Ultimate Rejection? A Psychological Study. Buckingham: Open University Press (1996).

[10] Catechismo della Chiesa Cattolica. Compendio. Roma: Libreria Editrice Vaticana (2005). Available from: http://www.vatican.va/archive/compendium_ccc/documents/archive_2005_compendium-ccc_it.html

[11] Steinberg A. Risky treatments: a Jewish medical ethics perspective. Rambam Maimonides Med J (2015) 6(3):1–6. 10.5041/RMMJ.10217 26241221 PMC4524405

[12] Bathia MS Stigma, suicide and religion. Br J Psychiatry (2002) 180:188–9. 10.1192/bjp.180.2.188-a 11823337

[13] Dervick K Ocquendo MA Grunembaum MF Ellis S Burke AK Mann JJ. Religious affiliation and suicide attempt. Am J Psychiatry (2004) 16:2303–8. 10.1176/appi.ajp.161.12.2303 15569904

[14] Dervick K Carballo JJ Baca-Garcia E Galfalvy HC Mann JJ Brent A Moral or religious objections to suicide may protect against suicidal behaviour in bipolar disorder. J Clin Psychiatry (2011) 72:1390–6. 10.4088/JCP.09m05910gre 21367349 PMC3785100

[15] Sudak H Maxim H Carpenter M. Suicide and stigma; a review of the literature and personal reflections. Acad Psychiatry (2008) 32:136–42. 10.1176/appi.ap.32.2.136 18349334

[16] Witte TK Smith AR Joiner TE Jr Reason for cautious optimism? Two studies suggesting reducing stigma against suicide. J Clin Psychol (2010) 66:611.626 10.1002/jclo.20691 20455251 PMC3308355

[17] Battermann PJ Calear AL Christensen H. The stigma of suicide scale. Psychometric properties and correlates of the stigma of suicide. Crisis (2013) 34:13–23. 10.1027/0227-5910/a000156 22846447

[18] Pompili M Stigma and suicide risk. In: Tatarelli R Pompili G Giradi P, editors. Suicide in Schizophrenia. Hauppauge, NY: Nova Biomedical Books (2007). p. 329–36.

[19] Oliffe JL Ogrodniczuk JS Gordon SJ Creighton G Kelly MT Black N Stigma in male depression and suicide: a Canadian sex comparison study. Community Ment Health J (2016) 52:302–10. 10.1007/s10597-015-9986-x 26733336 PMC4805721

[20] Batterham PJ Alear AL Christensen H. Correlates of suicide stigma and suicide literacy in the community. Suicide Life Threat Behav (2013) 43:406–17. 10.1111/sltb.12026 23556504

[21] Park S Kim MJ Cho MJ Lee JY Factors affecting stigma towards suicide and depression: a Korean nationwide study. Int J Soc Psychiatry (2015) 61:811–7. 10.1177/0020764015597015 26228237

[22] Li A Huang X Hao B O’Dea B Christensen H Zhu T. Attitudes towards suicide attempts broadcast on social media: an exploratory study of Chinese microblogs. Peer J (2015) 8:e1209. 10.7717/peerj.1209 26380801 PMC4570843

[23] Sand E Gordon KH Bresin K The impact of specifying suicide as the cause of death in an obituary. Crisis (2015) 34:63–6. 10.1027/0227-2910/a000154 22846446

[24] Sheehan LL Corrigan PW Al-Khouja MA Stigma of Suicide Research Team Stakeholder perspectives on the stigma of suicide attempt survivors. Crisis (2016) 26:1–9. 10.1027/0227-5910/a000413 27561224

[25] Frey LM Hans JD Cerel J. Perceptions of suicide stigma. Crisis (2016) 37:95–103. 10.1027/0227-5910/a000358 26695868

[26] Cvinar JG. Do suicide survivors suffer social stigma: a review of the literature. Perspect Psychiatr Care (2005) 41:14–21. 10.1111/j.0031-5990.2005.00004.x 15822848

[27] Pitman AL Osborn DP Rantell K King MB. The stigma perceived by people bereaved by suicide and other sudden deaths: a cross-sectional UK study of 3432 bereaved adults. J Psychosom Res (2016) 87:22–9. 10.1016/j.jpsychores.2016.05.009 27411748 PMC4988532

[28] Kalish RA Social distance and dying. Community Ment Health J (1966) 2:152–5. 10.1007/BF01420690 24190772

[29] Lester D The stigma against dying and suicidal patients: a replication of Richard Kalish study twenty-five years later. Omega J Death Dying (1993) 26:71–5. 10.2190/PB36-AUG6-1R77-LPMG

[30] Pompili M Shrivastava A Serafini G Innamorati M Milelli M Erbuto DM Bereavement after the suicide of a significant other. Indian J Psychiatry (2013) 55:256–63. 10.4103/0019-5545.117145 24082246 PMC3777347

[31] Sween C Walby FA Suicide survivors mental health and grief reactions: a systematic review of controlled studies. Suicide Life Threat Behav (2008) 38:13–29. 10.1521/suli.2008.38.1.13 18355105

[32] Scocco P Castriotta C Toffol E Preti A Stigma of suicide attempt (STOSA) and stigma of suicide and suicide survivor (STOSASS) scale: two new assessment tools. Psychiatry Res (2012) 200:872–8. 10.1016/j.psychres.2012.06.033 22819276

[33] Wicklander M Shame reactions after suicide attempts. Scand J Caring Sci (2003) 17:293–300. 10.1046/j.1471-6712.2003.00227.x 12919465

[34] Wolk Wassermann D. The intensive care unit and the suicide attempt patient. Acta Psychiatr Scand (1985) 71:581–95. 10.1111/j.1600-0447.1985.tb02552.x 4024974

[35] Scocco P Toffol E Preti A SOPRoxi Project Team. Psychological distress increases perceived stigma toward attempted suicide among those with a history of past attempted suicide. J Nerv Ment Dis (2016) 204:194–202. 10.1097/NMD0000000000000457 26751731

[36] Pitman AL Osborn D King MB Erlangsen A. Effects of suicide bereavement on mental health and suicide risk. Lancet Psychiatry (2014) 1:186–98. 10.1016/S2215-0366(14)70224-X 26360405

[37] Pitman AL Osborn DPJ Rantell K King MB. Bereavement by suicide as a risk factor for suicide attempt: a cross-sectional national UK-wide study of 3432 young bereaved adults. BMJ Open (2016) 6(1):e009948. 10.1136/bmjopen-2015-009948 26813968 PMC4735143

[38] Eagles JM Carson DP Begg A Naji SA Suicide prevention: a study of patient’s view. Br J Psychiatry (2003) 182:261–5. 10.1192/bjp.182.3.261 12611791

[39] Pompili M Mancinelli I Tatarelli R Stigma as a cause of suicide. Br J Psychiatry (2003) 183:173–4. 10.1192/bjp.183.2.173-a 12893678

[40] Assefa D Shibre T Aher L Fekadu A Internalized stigma among patients with schizophrenia in Ethippia: a cross-sectional facility-based study. BMC Psychiatry (2012) 12:239 10.1186/1471-244-X-12-239 23272796 PMC3544560

[41] Uçok A Karadayi G Emiroglu B Sartorius N. Anticipated discrimination is related to symptom severity, functionality and quality of life in schizophrenia. Psychiatry Res (2013) 209:333–9. 10.1016/psychres.2013.02.022 23528519

[42] Latalova K Prasko J Kamaradova D Ociskova M Cinculova A Grambal A Self-stigma and suicidality in in patients with neuroctic-spectrum disorders: a cross sectional study. Neuroendocrinol Lett (2014) 35:474–80. 25433850

[43] Farrelly S Jeffery D Rush N Williams P Thornicroft G Clement S The link between mental-health discrimination and suicidality: service user perspectives. Psychol Med (2015) 45:1013–22. 10.1017/S0033291714003158 25678059

[44] Yoo T Kim SW Kim SY Lee JY Kang HJ Bae KY Relationship between suicidality and low self-esteem in patients with schizophrenia. Clin Psychopharmacol Neurosci (2015) 13:296–301. 10.9758/cpn.2015.13.3.296 26598589 PMC4662169

[45] Campo-Arias A Herazo E The stigma-discrimination complex associated with mental disorder as a risk fator for suicide. Rev Colomb Psiquiatr (2015) 44:243–50. 10.106/j.rcp.2015.04.003 26578476

[46] Oexle N Waldmann T Staiger T Xu Z Rush N Mental illness stima: the role of public and individual stigma. Epidemiol Psychiatr Sci (2016) 6:1–7. 10.1017/S2045796016000949 PMC6998948 27919303

[47] Xu Z Muller M Heekeren K Theodoridou A Metzler S Dvorsky D Pathways between stigma and suicidal ideation among people at risk of psychosis. Schizophr Res (2016) 172:184–8. 10.1016/j.schres.2016.01.048 26843510

[48] Schomerus G Evans-Lacko S Rüsch N Mojtabai R Angermeyer MC Thornicroft G. Collective levels of stigma and national suicide rates in 25 European countries. Epidemiol Psychiatr Sci (2015) 24:166–71. 10.1017/S2045796014000109 24576648 PMC6998117

[49] Rush N Zlati A Black G Thornicroft G Does the stigma of mental illness contributes to suicidality? Br J Psychiatry (2014) 205:257–9. 10.1192/bjp.bp.114.145755 25274313

[50] Oexle N Rush N Viering S Wyss C Seifritz E Xu Z Self-stigma and suicidality: a longitudinal study. Eur Arch Psychiatry Clin Neurosci (2016). 10.1007/s00406-016-0698-1 27169427

[51] Sharaf AY Ossman LH Lachine OA A cross sectional study of the relationship between illness insight, internalized stigma and suicidal risk in individuals with schizophrenia. Int J Nurs Stud (2012) 49:1512–20. 10.1016/j.ijnurstu.2012.08.006 22939218

[52] Yoo T Kim SW Kim SY Lee SY Hang HJ Bae KY Relationship between suicidality and low self-esteem in patients with schizophrenia. Clin Psychopharmacol Neurosci (2015) 13:296–301. 10.9758/cpn.2015.13.3.296 26598589 PMC4662169

[53] Lehmann M Hilimire MR Yang LH Link BG DeVylder JE. Investigating the relationship between self-esteem and stigma among young adults with history of suicide attempts. Crisis (2016) 4:1.6. 10.1027/0227-5910/a000399 27338292

[54] Xu Z MÜller M Heekeren K Theodoridou A Metzler S Dvorsky D Pathways between stigma and suicidal ideation among people at risk of psychosis. Schizophr Res (2016) 172:184–8. 10.1016/j.schres.2016.01048 26843510

[55] Oexle N Ajdacic-Gross V Kilian R Müller M Rodgers S Xu Z Mental illness stigma, secrecy and suicidal ideation. Epidemiol Psychiatr Sci (2017) 26:53–60. 10.1017/S2045796015001018 26606884 PMC6998646

[56] Griffiths KM Crisp DA Jorm AF Christensen H. Does stigma predict a belief in dealing with depression alone? J Affect Disord (2011) 132:413–7. 10.1016/j.jad.2011.03.012 21440305

[57] Schwenck TL Davis L Wimsatt LA. Depression, stigma and suicidal ideation in medical students. JAMA (2010) 304:1181–90. 10.1001/jama.2010.1300 20841531

[58] Dyrbiye LN Eacker A Durning SJ Brazeau C Moutier C Massie C The impact of stigma and personal experiences on the help-seeking behavior of medical students with burnout. Acad Med (2015) 90:61–9. 10.1097/ACM.0000000000000655 25650824

[59] Arria AM Winick ER Garbier-Dykstra LM Vincent KB Caldeira KM Wilcox HC Help seeking and mental health service utilization among college students with a history of suicide ideation. Psychiatr Serv (2011) 62:1510–3. 10.1176/appi.ps.005562010 22193801 PMC3246367

[60] Downs MF Eisenberg D. Help seeking and treatment use among suicidal college students. J Am Coll Health (2012) 60:104–14. 10.1080/07448481.2011.619611 22316407

[61] Czyz EK Horwitz AG Eisenberg D Kramer A King CA. Self-reported barriers to professional help seeking among college students at elevated risk for suicide. J Am Coll Health (2013) 61:398–406. 10.1080/07448481.2013.820731 24010494 PMC3788673

[62] Luoma JB Martin CE Pearson JL Contact with mental health and primary care providers before suicide: a review of evidence. Am J Psychiatry (2002) 159:909–901. 10.1176/appi.ajp.159.6.909 12042175 PMC5072576

[63] Henderson C Evans-Lacko S Thornicroft G. Mental illness stigma, help seeking and public health programs. Am J Public Health (2013) 103:777–80. 10.2105/AJPH.2012.301056 23488489 PMC3698814

[64] Bruffarerts R Demyttenaere K Hwang I Chiu WT Sampson N Kessler RC Treatment of suicidal people around the world. Br J Psychiatry (2011) 199:64–70. 10.1192/bjp.bp.110.084129 21263012 PMC3167419

[65] Reynders A Kerkhof AJ Molenberghs G Van Audenhove C Attitudes and stigma in relation to help-seeking intentions for psychological problems in low and high suicide rates regions. Soc Psychiatry Psychiatr Epidemiol (2014) 49:231–9. 10.1007/s00127-013-0745 23896893

[66] Ausberger A Yeung A Dougher M Hahm HC. Factors influencing the underutilization of mental health services among Asian American women with a history of depression and suicide. BMC Health Serv Res (2015) 15:542. 10.1186/s12913-015-1191-7 26645481 PMC4673784

[67] Perez-Rodriguez MM Baca-Garcia E Oquendo MA Wang S Wall MM Liu SM Relationship between acculturation, discrimination and suicidal ideation and attempts among US Hispanics in the National Epidemiologic Survey of Alcohol and related conditions. J Clin Psychiatry (2014) 75:399–407. 10.4088/JCP.13m08548 24813407

[68] Meyer IH. Prejudice, social stress and mental health in lesbian, gay and bisexual populations: conceptual issues and research evidence. Psychol Bull (2003) 129:674–97. 10.1037/0033-2909.129.5.674 12956539 PMC2072932

[69] Warner J McKeown E Griffin M Johnson K Ramsay A Cort C Rates and predictors of mental illmess in gay men, lesbians and bisexual men and women: results from a suvrey based in England and wales. Br J Psychiatry (2004) 185:479–85. 10.1192/bjp.185.6.479 15572738

[70] Hidaka Y Operario D Attempted suicide, psychological health and exposure to harassment among Japanese homosexual, bisexual of other men questioning their sexual orientation recruited via the internet. J Epidemiol Community Health (2006) 60:962–7. 10.1136/jech.2005.045336 17053285 PMC2465476

[71] Cochran SD Mays VM Alegria M Ortega AN Takeuci D Mental health and substance use disorders among Latino and Asian American lesbian, gay, and bisexuals adults. J Consult Clin Psychol (2007) 75:785–94. 10.1037/0022-006X.75.5.785 17907860 PMC2676845

[72] King M Semlyen J Tay SS Killaspy H Osborn D Popelyuk D A systematic review of mental disorder, suicide, and deliberate self harm in lesbian, gay and bisexual people. BMC Psychiatry (2008) 8:70. 10.1186/1471-244X-8-70 18706118 PMC2533652

[73] Newcomb ME Mustanski B. Internalized homophobia and internalizing mental health problems: a meta-analytic review. Clin Psychol Rev (2010) 30:1019–29. 10.1016/j.cpr.2010.07.003 20708315

[74] Chakraborty A McManus S Brugha TS Bebbington P King M Mental health and the non-heterosexual population in England. Br J Psychiatry (2011) 198:143–8. 10.1192/bjp.bp.110.082271 21282785

[75] Baiocco R Ioverno S Cerutti R Santamaria F Fontanesi L Lingiardi V Suicidal ideation in Spanish and Italian lesbian and gay young adults: the role of internalized sexual stigma. Psicothema (2014) 26:490–6. 10.7334/psicothema2014.1 25340896

[76] Hatzenbuehler ML Bellatorre A Lee Y Finch BK Muennig P Fiscella K. Structural stigma and all-cause mortality in sexual minority populations. Soc Sci Med (2014) 103:33–41. 10.1016/j.socscimed.2013.06.005 23830012 PMC3818511

[77] Lea T de Wit J Reynolds R Minority stress in lesbian, gay, and bisexual young adults in Australia: associations with psychological distress, suicidality, and substance use. Arch Sex Behav (2014) 43:1571–8. 10.1007/s10508-014-0266-6 24573397

[78] Pompili M Lester D Forte A Seretti ME Erbuto D Lamis DA Bisexuality and suicide: a systematic review of the current literature. J Sex Med (2014) 11:1903–13. 10.1111/jsm.12581 24839908

[79] Skerrett DM Kolves K De Leo D Are LGBT populations at higher risk for suicidal behaviors in Australia? Research findings and implications. J Homosex (2015) 62:833–901. 10.1080/00918369.2014.1003009 25569508

[80] Miller LR Grollman EA. The social costs of gender nonconformity for transgender adults: implications for discrimination and health. Sociol Forum (Randolph N J) (2015) 30:809–31. 10.1111/socf.12193 27708501 PMC5044929

[81] Perez-Brumer A Hatzenbuehler ML Oldenburg CE Bockting W. Individual- and structural-level risk factors for suicide attempts among transgender adults. Behav Med (2015) 41:164–71. 10.1080/08964289.2015.1028322 26287284 PMC4707041

[82] Kim S Yang E. Suicidal ideation in gay men and lesbians in South Korea: a test of the interpersonal-psychological model. Suicide Life Threat Behav (2015) 45:98–110. 10.1111/sltb.12110 25220014

[83] Lehavot K Simpson TL Shiperd JC. Factors associated with suicidality among a national sample of transgender veterans. Suicide Life Threat Behav (2016) 46:507–24. 10.1111/sltb.12233 26878597

[84] Marshall BD Socias ME Kerr T Zalazar V Sued O Aristequi I. Prevalence and correlates of lifetime suicide attempts among transgender persons in Argentina. J Homosex (2016) 63:955–67. 10.1080/00918369.2015.1117898 26566683

[85] Cho B Sohn A. How do sexual identity, and coming out affect stress, depression and suicidal ideation and attempts among men who have sex with men in South Korea? Osong Public Health Res Perspect (2016) 7:281–8. 10.1016/j.phrp.2016.09.001 27812485 PMC5079205

[86] Tebbe EA Moradi B. Suicide risk in trans populations: an application of minority stress theory. J Couns Psychol (2016) 63:520–33. 10.1037/cou0000152 27089059

[87] Swannell S Martin G Page A. Suicidal ideation, suicide attempts and non-suicidal self-injury among lesbian, gay, bisexual and heterosexual adults: findings from an Australian national study. Aust N Z J Psychiatry (2016) 50:145–53. 10.1177/0004867415615949 26631718

[88] Cotè TR Biggar RJ Dannenberg AL Risk of suicide among persons with AIDS. A national assessment. JAMA (1992) 268:2066–8. 1404744 10.1001/jama.1992.03490150118035

[89] Vance DE Moneyham L Fordham P Struzick TC. A model of suicidal ideation in adults aging with HIV. J Assoc Nurses AIDS Care (2008) 19:375–84. 10.1016/j.jana.2008.04.011 18762145

[90] Wu YL Yang HY Wang J Yao H Zhao X Chen J Prevalence of suicidal ideation and associated factors among HIV-positive MSM in Anhui, China. Int J STD AIDS (2015) 26:496–502. 10.1177/0956462414544722 25060699

[91] Bitew H Andargie G Tadesse A Belete A Fekadu W Mekonen T. Suicidal ideation, attempt and determining factors among HIV/AIDS patients, Ethiopia. Depress Res Treat (2016) 2016:8913160. 10.1155/2016/8913160 27747101 PMC5055996

[92] Chen EY Fettich KC McCloskey MS Correlates of suicidal ideatin and/or behavior in bariatric-surgery-seeking individuals with severe obesity. Crisis (2012) 33:137–43. 10.1027/0227-5910/a000115 22343060

[93] Levy BR Pilver CE. Residual stigma: psychological distress among the formerly overweight. Soc Sci Med (2012) 75:297–9. 10.1016/socscimed.2012.03.007 22560867 PMC3711678

[94] Ramos Salas X. The ineffectiveness and unintended consequences of the public health war on obesity. Can J Public Health (2015) 106(2):e79–81. 10.17269/cjph.106.4757 25955676

[95] Rumrill P Fitzgerald SM Using Narrative Literature Reviews to Build a Scientific Knowledge Base, Research Methodology. (2008). Available from: https://doresearch.wordpress.com/category/literature-review/12441470

[96] Mehta N Clement S Marcus E Stona AC Bezborodovs N Evans-Lacko S Evidence for effective interventions to reduce mental health-related stigma and discrimination in the medium and long term: systematic review. Br J Psychiatry (2015) 207:377–84. 10.1192/bjp.bp.114.151944 26527664 PMC4629070

[97] Thornicroft G Mehta N Clement S Evans-Lacko S Doherty M Rose D Evidence for effective interventions to reduce mental-health-related stigma and discrimination. Lancet (2016) 387(10023):1123–32. 10.1016/S0140-6736(15)00298-6 26410341

[98] Calear AL Batterham PJ Christensen H. Predictors of help-seeking for suicidal ideation in the community: risks and opportunities for public suicide prevention campaigns. Psychiatry Res (2014) 219:525–30. 10.1016/j.psychres.2014.06027 25048756

[99] Batterham PJ Calear AL Christensen H. Correlates of suicide stigma and suicide literacy in the community. Suicide Life Threat Behav (2013) 43:406–17. 10.1111/sltb.12026 23556504

[100] Clement S Lassman F Barley E Evans-Lacko S Williams P Yamaguchi S Mass media interventions for reducing mental health-related stigma. Cochrane Database Syst Rev (2013) 23(7):CD009453. 10.1002/14651858.CD009453.pub2 23881731 PMC9773732

[101] Sisask M Varnick A. Media roles in suicide prevention: a systematic review. Int J Environ Res Public Health (2012) 9:123–38. 10.3390/ijerph9010123 22470283 PMC3315075

